# MyCARdiac score: integrating cardiac imaging and biomarkers to predict outcomes in RRMM patients receiving cilta-cel

**DOI:** 10.1038/s41375-026-02887-5

**Published:** 2026-02-17

**Authors:** Thomas C. Wiemers, Nora Grieb, Maximilian Ferle, Michael Rade, David Fandrei, Patrick Born, Luise Fischer, Song-Yau Wang, Sabine Seiffert, Anja Grahnert, Maik Friedrich, Ronny Baber, Markus Kreuz, Klaus H. Metzeler, Marco Herling, Carmen D. Herling, Madlen Jentzsch, Georg-Nikolaus Franke, Andreas Boldt, Thomas Neumuth, Ulrike Köhl, Kristin Reiche, Tina Stegmann, Timm Denecke, Uwe Platzbecker, Vladan Vučinić, Hans-Jonas Meyer, Daniel Lavall, Maximilian Merz

**Affiliations:** 1 Department of Visceral, Transplantation, Vascular and Thoracic Surgery, University Hospital Leipzig, Leipzig, Germany; 2 Department of Hematology, Hemostaseology and Cellular Therapy, University Hospital Leipzig, Leipzig, Germany; 3 Innovation Center Computer Assisted Surgery (ICCAS), University Hospital Leipzig, Leipzig, Germany; 4 Fraunhofer Institute for Cell Therapy and Immunology IZI, Leipzig, Germany; 5 Center for Scalable Data Analytics and Artificial Intelligence (ScaDS.AI), University of Leipzig, Leipzig, Germany; 6 Institute of Clinical Immunology, Medical Faculty, University of Leipzig, Leipzig, Germany; 7 Institute for Laboratory Medicine Clinical Chemistry and Molecular Diagnostics, University Hospital Leipzig, Leipzig, Germany; 8 Leipzig Medical Biobank, Medical Faculty, University of Leipzig, Leipzig, Germany; 9 Department of Cardiology, University Hospital Leipzig, Leipzig, Germany; 10 Department of Diagnostic and Interventional Radiology, University Hospital Leipzig, Leipzig, Germany; 11 Myeloma and Cellular Therapy Services, Memorial Sloan Kettering Cancer Center, New York, NY USA

**Keywords:** Myeloma, Myeloma, Risk factors, Disease-free survival, Medical imaging

## To the Editor

Chimeric antigen receptor (CAR) T-cell therapy has transformed treatment for relapsed and refractory multiple myeloma (RRMM), but its wider use has renewed attention to off-target toxicities. While cytopenias, infections and immune-related adverse events such as cytokine release syndrome (CRS) and Immune Effector Cell-Associated Neurotoxicity Syndrome (ICANS) have been well described [1–3], less is known about the prognostic implications of baseline cardiac status in patients undergoing CAR T-cell therapies. A prospective, multi-construct cohort found that structural measures like increased interventricular septal wall thickness (IVSD) and reduced left ventricular ejection fraction (LVEF) together with early troponin rises, were associated with worse outcomes, supporting the value of structural and biomarker signals for risk stratification [4]. Real-world data with idecabtagene vicleucel (Ide-cel) and ciltacabtagene autoleucel (Cilta-cel) also reported adverse cardiac events, including heart failure, atrial fibrillation, and ventricular arrhythmia following anti B-cell maturation antigen (BMCA) CAR T-cell therapy [5, 6]. Despite the growing body of evidence regarding the distinct toxicity profile of Cilta-cel compared to Ide-cel [7], evidence linking baseline cardiac conditions to outcomes or tumor burden remains scarce. Here, we characterize baseline cardiac profiles in patients treated with Cilta-cel and introduce the MyCARdiac score, a composite of routine echocardiographic, computed tomography (CT), and NT-proBNP measures, to improve baseline risk stratification and to guide post-infusion surveillance.

We analyzed 72 consecutive Cilta-cel–treated patients (2021–2025, University Hospital Leipzig) with written informed consent and ethics approval (361/22-ek). Baseline cardiovascular profiles, including risk factors and cardiac comorbidities were extracted from electronic medical records. Post-infusion cardiac events included hospitalization for cardiac causes, worsening heart failure, or cardiovascular death. CRS and ICANS were graded per ASTCT criteria [1]. The Revised International Staging System (R-ISS) and log_2_ transformed Endothelial Activation and Stress Index (EASIX) scores, as well as the presence of extramedullary disease (EMD) were assessed as described previously [8, 9]. Immune effector cell-associated hematotoxicity (ICAHT) was graded separately for absolute neutrophil (N-ICAHT) and platelet count (T-ICAHT), applying published criteria [2, 3]. All patients underwent structured transthoracic echocardiographic evaluation as part of the pre-treatment workup within 30 days prior to CAR T-cell infusion using commercially available ultrasound systems (GE-Vivid-E95 and GE-Echopac-Software). Assessed parameters included LVEF, IVSD, left posterior wall diastolic thickness (LVPWd), vena cava (VC)-collapsibility as well as body surface-indexed left ventricular end-diastolic and end-systolic diameters (LVDd, LVDs), left ventricular mass index (LVM-Index), left atrial diameter (LADs), and global longitudinal strain (GLS) following current guidelines [10]. Coronary artery calcification (CAC) was assessed on the most recent pre-treatment CT scans using the Weston-scoring method (range 0–12) [11]. NT-proBNP was measured in median 6 (range 0–97) days before CAR-T cell infusion. The Heart Failure Association-International Cardio-Oncology Society (HFA-ICOS) score was calculated for comparison with other cardio-oncology risk assessments [12].

Peripheral blood samples were collected at lymphodepletion (LP), CAR T-cell infusion (day 0), and on days 7, 14, 30, and 100 post-infusion. These samples were then used to measure soluble BCMA (sBCMA) and toidentify bystander T-cells as well as different subsets of CAR T-cells, as described previously [13]. Statistical analyses were conducted using R version 4.3.0. Progression-free survival (PFS) and overall survival (OS) were defined as the time from CAR T-cell infusion to relapse or death from any cause, respectively.

The median age at the time of CAR T-cell therapy was 65.3 years (range 31.9–81.6), with 34 (47.2%) males and 38 (52.8%) females. Median Follow-up time was 6 (1–40) months. The median number of prior therapies was 7 (range 1–17). Preexisting cardiac disease was observed in 23 (31.9%) patients, including heart failure in 12 (16.7%), coronary artery disease in 3 (4.2%), valvular heart disease in 3 (4.2%), atrial fibrillation in 11 (15.2%), and preexisting cardiomyopathy in 2 (2.8%) patients. One patient had a history of myocarditis. Cardiac risk factors were present in 45 (62.5%) patients. Hypertension was the most common, affecting 29 (40.3%) patients, followed by smoking in 11 (15.3%) and diabetes in 6 (8.3%). Importantly, none of these baseline characteristics was linked to the occurrence of CRS/ICANS, cardiac adverse events and worse PFS following CAR T-cell therapy (*p* > 0.05, respectively). During follow-up 4 (5.6%) patients developed adverse cardiac events.

**Fig. 1:**
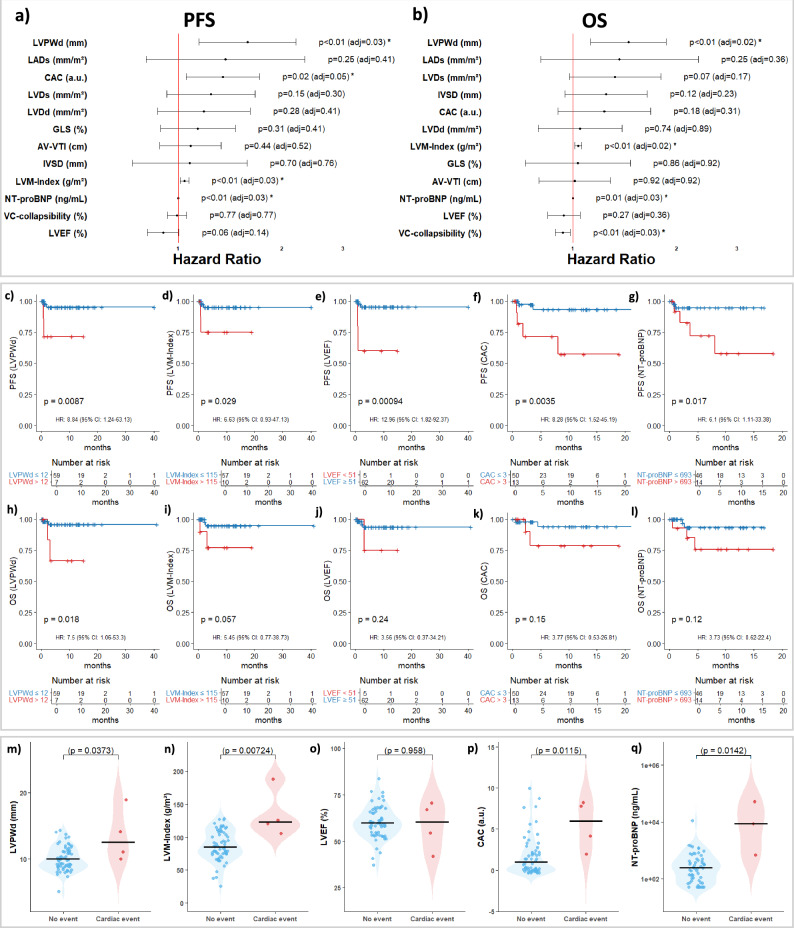
I Association of echocardiographic and cardiac biomarkers with survival outcomes after Cilta-cel. Univariate Cox regression analyses of echocardiographic parameters and cardiac biomarkers with respect to progression-free survival (PFS, **a**) and overall survival (OS, **b**). Hazard ratios with 95% confidence intervals (95% CI) are shown. Raw *p*-values and false discovery rate (FDR)-adjusted *p*-values are reported, with statistical significance indicated by asterisks based on the adjusted *p*-values (*p* ≤ 0.05, *p* < 0.01). **c–g** Kaplan–Meier curves of PFS stratified by optimized cut-off values for LVPWd, LVM-Index, LVEF, CAC, and NT-proBNP. **h–l** Kaplan–Meier curves of OS stratified by the same parameters. HR, 95% CI, and log-rank p-values are shown, and numbers at risk are indicated below each plot. **m–q** Box plots showing the associations of the same parameters with the occurrence of adverse cardiac events. The Mann–Whitney U test was used for statistical analysis, and statistical significance was defined as *p* < 0.05.

To assess the effect of baseline echocardiographic parameters, CAC score as well as NT-proBNP measures on survival, we fitted univariate Cox proportional-hazards models for PFS and OS using all variables as continuous parameters (Fig. 1a, b). To account for multiple comparisons, *p*-values were adjusted using the false discovery rate (FDR) method. Both the raw *p*-values and the FDR-adjusted *p*-values (*p*_adj_) are reported. Among echocardiographic variables, greater LVPWd (*n* = 67) was associated with worse PFS (HR:1.59, 95% CI: 1.15–2.19, *p* = 0.005, *p*_adj_ = 0.03) and OS (HR: 1.45, 95% CI: 1.13–1.87, *p* = 0.004, *p*_adj_ = 0.02). Higher LVM-Index (*n* = 68) likewise predicted shorter PFS (HR: 1.04, 95% CI: 1.01–1.07, *p* = 0.008, *p*_adj_ = 0.03) and OS (HR: 1.03, 95% CI: 1.01–1.06, *p* = 0.003, *p*_adj_ = 0.02). Reduced VC-collapsibility (*n* = 57) was associated with shorter OS (HR: 0.93, 95% CI: 0.89–0.98, *p* = 0.007, *p*_adj_ = 0.03). Lower baseline LVEF (*n* = 68) showed a trend towards worse PFS (HR: 0.90, 95% CI: 0.81–1.00, *p* = 0.058, *p*_adj_ = 0.14). Higher CAC (*n* = 64) scores were linked to reduced PFS (HR: 1.35, 95% CI: 1.05–1.72, *p* = 0.017, *p*_adj_ = 0.05) and elevated NT-proBNP (*n* = 61) to shorter PFS (HR: 1.08, 95% CI: 1.02–1.14, *p* = 0.005, *p*_adj_ = 0.03) and OS (HR:1.06, 95% CI:1.014–1.105, *p* = 0.01, *p*_adj_ = 0.03).

Optimized cut-off values for each variable identified in univariate survival analyses were determined using maximally selected rank statistics for PFS. These thresholds were then applied to categorize patients for PFS and OS analyses (Fig. 1c–l). The cut-offs were 12 mm for LVPWd, 115 g/m^2^ for LVM-Index, 3 for CAC-score and 693 ng/mL for NT-proBNP with values above being considered as high. LVEF values below 51% were classified as low. For PFS, higher LVPWd (Fig. 1c), increased LVM-Index (Fig. 1d), reduced LVEF (Fig. 1e), elevated CAC scores (Fig. 1f) as well as increased NT-proBNP (Fig. 1g) were consistently linked with shorter PFS (*p*_logrank _< 0.05, respectively). Similarly, for OS, elevated LVPWd (*p*_logrank _= 0.018, Fig. 1h) and increased LVM-Index (*p*_logrank _= 0.057, Fig. 1i) were linked to adverse OS, while LVEF, CAC and NT-proBNP showed no association in OS analyses. Increased levels of LVPWd, LVM-Index, CAC-score, and NT-proBNP were associated with the occurrence of adverse cardiac events after CAR T-cell therapy (*p* < 0.05, respectively, Fig. 1m–q).

**Fig. 2:**
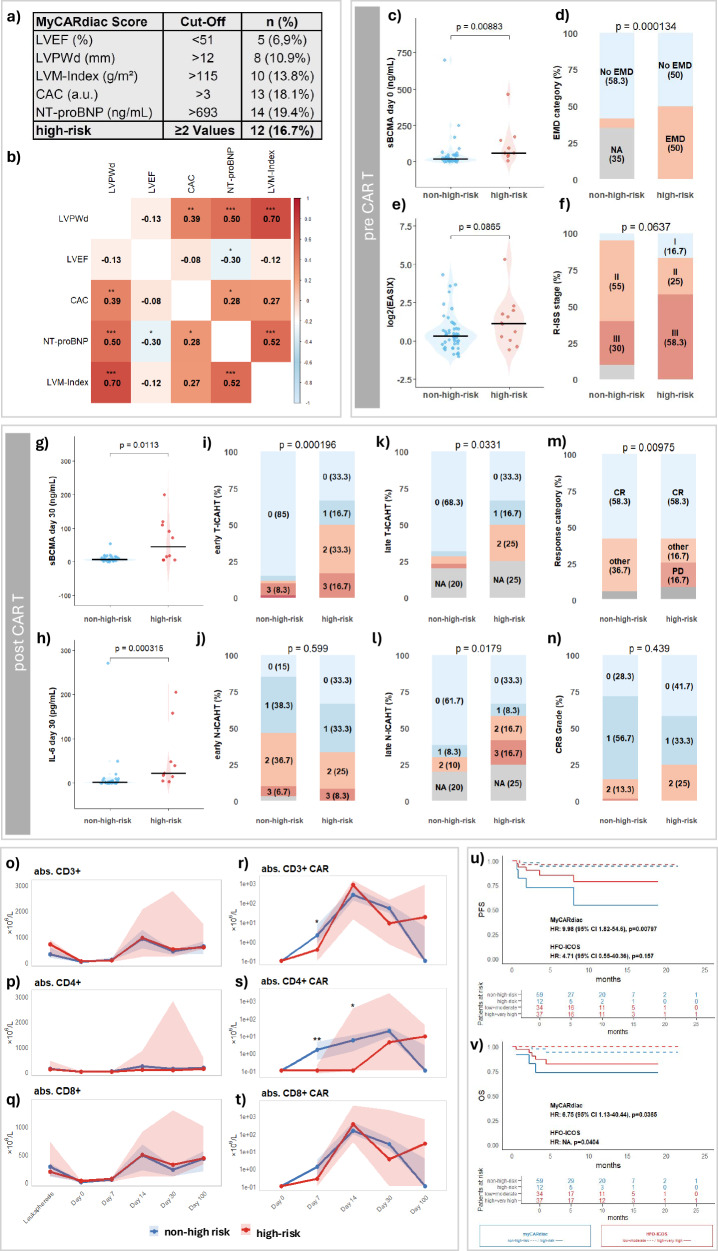
Impact of the MyCARdiac score and cardiovascular risk on treatment outcomes. **a** Definition of the MyCARdiac score components and distribution of high-risk patients. **b** Correlation matrix of echocardiographic parameters and cardiac biomarkers. **c–f** Baseline associations of MyCARdiac risk status with sBCMA, EMD, log_2_(EASIX), and R-ISS stage. **g**, **h** Post-infusion sBCMA and IL-6 levels by risk group. **i**–**l** Distribution of early and late N-ICAHT and T-ICAHT scores. **m**, **n** Association of MyCARdiac risk with treatment response and CRS grade. **o–t** Expansion kinetics of total CD3^+^, CD4^+^, and CD8^+^ T-cells and CAR^+^ subsets stratified by risk. **u**, **v** Kaplan–Meier curves for PFS and OS according to MyCARdiac (blue) and HFO-ICOS (red) risk classification. HR, 95% CI, and log-rank *p*-values are shown, and numbers at risk are indicated below each plot. Statistical significance was defined as *p* < 0.05. Two (**c**, **e**, **g**, **h**) or one (**o**–**t**) sided Mann-Whitney U, chi-square (**d**, **f**, **i**–**n**), and log-rank (**u**, **v**) tests were applied when appropriate.

To assess the impact of cardiac parameters on disease-related features in RRMM patients, we combined the cardiac parameters that were significantly associated with adverse outcomes into the MyCARdiac score (Fig. 2a). These variables showed high level of intercorrelation based on Spearman's coefficient analyses (Fig. 2b). Patients were classified as high-risk if at least two of the parameters exceeded their respective cut-off values. Notably, this classification was independent of age, body-mass-index, and renal function (*p* > 0.05, respectively).

Regarding parameters measured before CAR T-cell therapy we observed that high-risk patients had higher serum concentrations of sBCMA (*p* = 0.009, Fig. 2c) and EMD (*p* < 0.001, Fig. 2d). No significant differences were observed comparing the distribution of log_2_(EASIX) and R-ISS Scores (Fig. 2e, f).

Consistently, cardiac high-risk patients showed elevated sBCMA (*p* = 0.01, Fig. 2g) and IL-6 (*p* < 0.001, Fig. 2h) levels at day 30 post CAR T-cell infusion. This was accompanied by higher early (*p* < 0.001, Fig. 2i) and late (*p* = 0.03, Fig. 2k) T-ICAHT, as well as late N-ICAHT scores (*p* = 0.018, Fig. 2l), indicating greater depth and prolonged duration of thrombocytopenia and neutropenia. Similarly, best response differed between non-high- and high-risk patients with patients not responding to Cilta-cel being uniformly classified as high-risk (*p* = 0.01, Fig. 2m), while no difference was observed for CRS (Fig. 2n).

Next, we correlated non- and high-risk patients longitudinally to bystander T-cell abundancy from LP to day 100 after infusion without observing any differences between the groups (Fig. 2o–q). In contrast, high-risk patients showed significantly impaired CAR T-cell expansion for CAR CD3^+^ at day 7 (*p* < 0.05, Fig. 2r) and CAR CD4^+^ at days 7 and 14 (*p* < 0.05, Fig. 2s) without changes in CAR CD8^+^-cells irrespective of timepoint (Fig. 2t).

Finally, we applied the MyCARdiac score to survival analysis, where we observed reduced PFS (*p* = 0.008, Fig. 2u) and OS (*p* = 0.037, Fig. 2v) for patients that were classified as high-risk. In contrast, the HFA-ICOS assessment did not significantly stratify PFS (*p* = 0.157, Fig. 2u), while OS was reduced, consistent with the MyCARdiac score (*p* = 0.04, Fig. 2v).

Taken together, cardiac events were low in this cohort of RRMM patients receiving Cilta-cel treatment. Baseline markers of LV-remodeling (LVEF, LV-mass and -wall thickness), atherosclerosis (CAC-score), and subclinical congestion (NT-proBNP) provided independent prognostic information. Notably, NT-proBNP has been proposed as a predictive marker in MM [14]. Whether baseline remodeling reflects hypertension, intrinsic heart disease, or RRMM itself requires further research. The higher prevalence of EMD in high-risk compared to non-high-risk patients may indicate an association with RRMM rather than comorbidities. Cardiac MRI may play an important role in future studies to differentiate between entities of LV-hypertrophy [15]. Notably, patients with cardiac AL-amyloidosis were excluded from this study. The study is limited by its modest sample size, single-center setting and limited availability of baseline troponin measurements. Furthermore, adjudicated cardiotoxic events were infrequent, and risk associations largely reflect gradients in continuous cardiac measurements, which should be considered when interpreting the prognostic findings. We establish the first link between disease burden -measured by sBCMA, EMD, treatment response- and hematologic toxicities, captured by EASIX and ICAHT scores, with cardiac health in RRMM. Our derived MyCARdiac score identified a high-risk group with more aggressive disease features, impaired CAR T-expansion and inferior outcomes, showing better performance than HFA-ICOS for PFS. These findings support guideline-recommended routine cardiac assessment before CAR T-cell therapy to refine risk stratification. Future work should focus on external validation and confirm its applicability across broader patient populations.

## Data Availability

The data used in this study are available upon reasonable request to maximilian.merz@medizin.uni-leipzig.de.
